# Upregulation of microRNA-106b is associated with poor prognosis in hepatocellular carcinoma

**DOI:** 10.1186/s13000-014-0226-4

**Published:** 2014-12-03

**Authors:** Bin-Kui Li, Pin-Zhu Huang, Ji-Liang Qiu, Ya-Di Liao, Jian Hong, Yun-Fei Yuan

**Affiliations:** State Key Laboratory of Oncology in South China, Guangzhou, China; Department of Hepatobiliary Oncology, Sun Yat-sen University Cancer Center, 651 Dongfeng Road East, Guangzhou, Guangdong 510060 China

**Keywords:** Hepatocellular carcinoma, miR-106b, Prognosis

## Abstract

**Background:**

MicroRNA-106b (miR-106b) is a member of the miR-106b ~ 25 cluster. It has been reported that miR-106b acts as an oncogene and is upregulated in many human cancers. However, the prognostic value of miR-106b in hepatocellular carcinoma (HCC) remains unclear. The aim of this study was to investigate the clinical significance of miR-106b expression in HCC.

**Methods:**

We determined the expression level of miR-106b in 104 cases of paired HCC and adjacent non-tumor tissues by quantitative real-time PCR (qRT-PCR). The correlation between miR-106b expression and prognosis of HCC was studied by univariate and multivariate analysis. Multivariate analysis of the prognostic factors was performed with Cox proportional hazards model.

**Results:**

MiR-106b expression was significantly upregulated in as high as 76.0% of HCC tissues, compared with their non-tumor counterparts (*P* < 0.001). High miR-106b expression was significantly associated with large tumor size (*P* = 0.019) and vascular invasion (*P* = 0.016). Kaplan-Meier analysis showed that patients with high miR-106b expression had a worse overall survival than patients with low miR-106b expression (log-rank *P* = 0.004). The multivariate Cox regression analysis indicated that miR-106b expression was an independent prognostic factor for overall survival (HR, 2.002; 95% CI, 1.130-6.977; *P* = 0.027).

**Conclusion:**

Our data indicated that miR-106b expression was significantly upregulated in HCC and could serve as a potential unfavorable prognostic biomarker.

**Virtual Slides:**

The virtual slide(s) for this article can be found here: http://www.diagnosticpathology.diagnomx.eu/vs/13000_2014_226

## Background

Hepatocellular carcinoma (HCC) is one of the most common cancers worldwide with poor prognosis [1]. Accurate prediction of prognosis and patient stratification are crucial for guiding patients’ personalized clinical treatment. These are currently performed by clinical and/or pathological staging systems [2-4]. Recently important progress has been made with using comprehensive approaches to identify the molecular diversity in HCC. A large number of genetic and epigenetic abnormalities were found during the process of HCC development [5-8]. Consequently, many new prognostic biomarkers of HCC have been described [9,10]. Addition of new biomarkers in the current staging systems is likely to improve the prognostic prediction of HCC patients and identify specific subgroups of tumor.

MicroRNAs (miRNAs) are highly conserved, small non-coding RNA molecules of approximately 22 nucleotides in length that function as posttranscriptional gene regulators [11,12]. Many studies have shown that miRNAs are directly involved in cancer initiation and progression. Indeed, miRNAs could act as oncogenes or tumor suppressors [13,14]. In addition, some miRNA deregulations were found to be associated with specific tumor phenotype [15]. These observations suggest that miRNA could be used as putative biomarkers to classify tumors.

MiR-106b is a member of the miR-106b ~ 25 cluster, which consists of miR-106b, miR-93 and miR-25, that is encoded within intron 13 of the minichromosome maintenance complex component 7 (MCM7) gene on chromosome 7q22.1 [16]. MiR-106b has been reported as an oncogene and upregulated in many human cancers, including stomach, prostate and kidney [17-19]. Previous studies suggest that miR-106b also plays an important role in hepatocarcinogenesis [20-22]. In our previous study, using miRNA array, we also found miR-106b is one of the upregulated miRNAs in HCC [23]. However, the prognostic value of miR-106b in HCC has not been fully clarified yet.

In the present study, we investigated the expression levels of miR-106b in resected HCC specimens and adjacent non-tumor tissues. We also analyzed the association of miR-106b expression with clinicopathological characteristics and overall survival of the patients and determined whether miR-106b is potentially predictive of prognosis in HCC.

## Methods

### Patients and tissue samples

A total of 104 pairs of tumor and matched adjacent non-tumor liver tissues were collected from patients with HCC who underwent hepatic resections between July 2003 and July 2007 at Sun Yat-sen University Cancer Center. These patients were selected for hepatic resection in accordance with our defined criteria [24]. For all cases, tissue samples were obtained immediately after resection and were snap-frozen in liquid nitrogen and stored at −80°C until use. All tissues were archived in the institution’s liver tumor bank. The diagnosis was confirmed histologically in all cases, based on detailed examination of sections stained with H&E. None of the patients had received any other therapy, including percutaneous ablation and chemo-embolization, before surgery. The research protocol was approved by the Institutional Review Board of Sun Yat-sen University Cancer Center. Written informed consent was obtained from every patient to participate in this study.

### Follow-up

Clinical follow-up was performed for all the patients. All patients were monitored by physical examination, serum alpha-fetoprotein (AFP), ultrasonography, and x-ray every 1–3 months in the first year, and every 3–6 months thereafter for surveillance of recurrence or metastases. Computed tomography and/or magnetic resonance imaging and/or positron emission tomography were used whenever needed to confirm the clinical findings. The clinical end point in the present study was overall survival. Overall survival was defined as the interval from curative surgery to the date of death or the date of last contact if the patient was still alive. Survival information of all patients was updated by telephone visit and questionnaire letters.

### Quantitative real-time RT-PCR (qRT-PCR)

To detect miRNA expression by qRT-PCR, total RNA was extracted from tissue samples using the Trizol reagent (Invitrogen, Carlsbad, CA, USA). qRT-PCR of miR-106b was performed using Bulge-Loop™ miRNA qRT-PCR kits (Ribobio, Guangzhou, China) according to the manufacturer’s instructions. Briefly, reverse transcription was performed using a specific miR-106b stem-loop primer and the reverse primer for U6 small nuclear RNA, followed by real-time PCR with a miR-106b-specific forward primer and a universal reverse primer using an ABI-Prism 7900HT system (Applied Biosystems, Foster City, CA). All samples were carried out in triplicate. U6 was used as an endogenous control. The expression level of miR-106b for each sample was calculated, represented by the ΔCt value (Ct of miR-106b - Ct of U6). The relative miR-106b expression levels in paired tissues that were collected from the same patients were analyzed by the 2^-ΔΔCt^ method, represented by the -ΔΔCt value [−(ΔCt of tumor tissues - ΔCt of non-tumor tissues)]. The -ΔΔCt value represents the value of log_2_ T/NT for each patient.

### Statistical analysis

Statistical comparisons were performed using either the Student’s *t*-test, chi-square test or Fisher’s exact test, as appropriate. In a univariate analysis, survival curves were assessed by the Kaplan-Meier method, and differences between curves were analyzed using the log-rank test. Covariates with *P* values < 0.05 in the univariate analysis were subjected to multivariate analysis. Multivariate Cox regression models were constructed to estimate the hazard ratios (HRs) of independent factors for survival after controlling for potential confounding factors. All statistical analyses were carried out using the SPSS (Statistical Package for the Social Sciences) 13.0 software (SPSS, Chicago, IL) and GraphPad Prism 5.0 software (GraphPad Software, San Diego, CA). Two-tailed *P* values < 0.05 were considered statistically significant.

## Results

### MiR-106b expression in HCC

**Figure 1:**
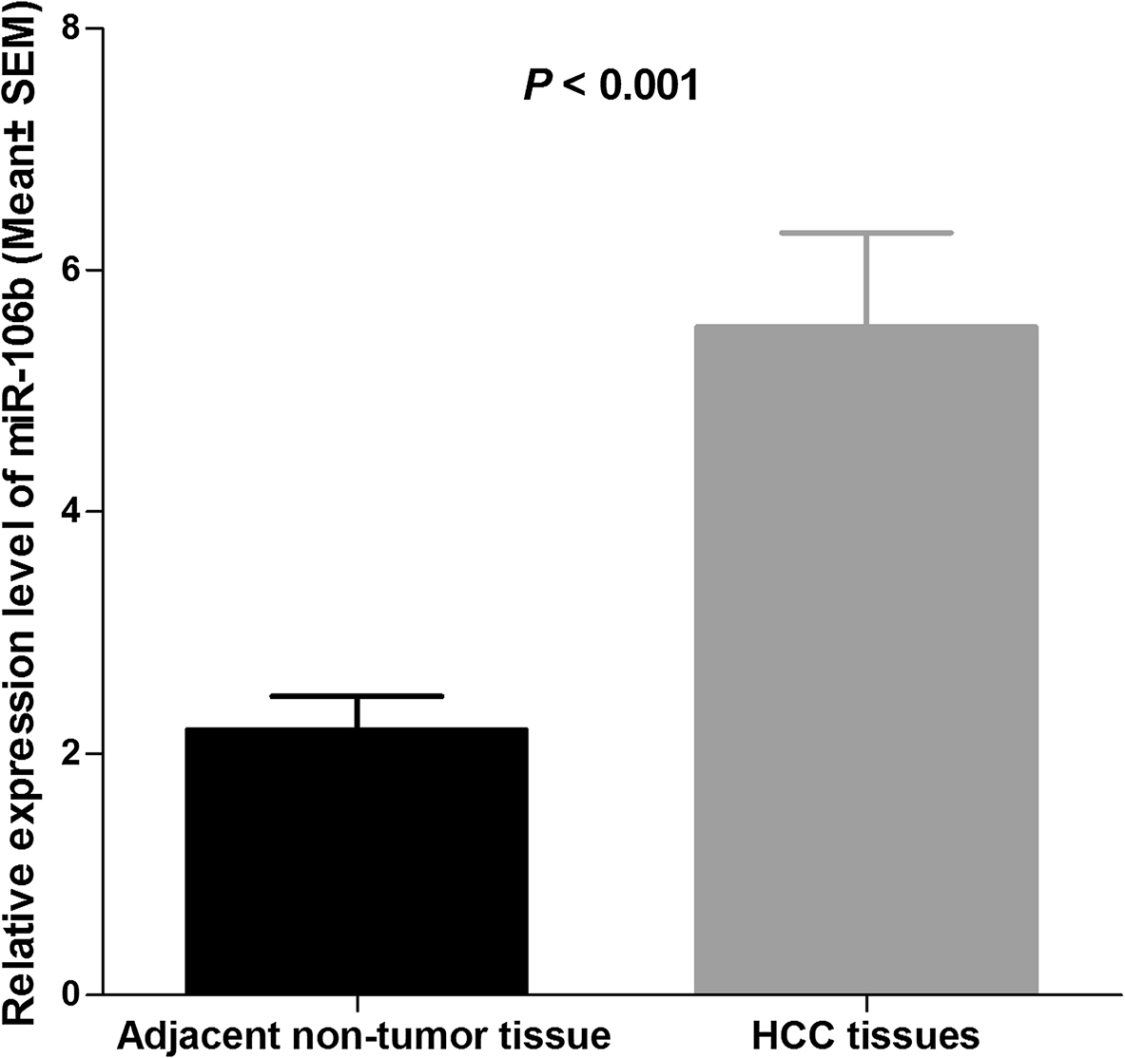
**Comparison of miR-106b expression levels between HCC tissues and adjacent non-tumor tissues.** Analysis using the Student’s t-test showed that the relative expression levels of miR-106b in the HCC tissues were significantly higher than those in adjacent non-tumor tissues (*P* < 0.001). qRT-PCR data are ratios to average of non-tumor tissues.

**Figure 2:**
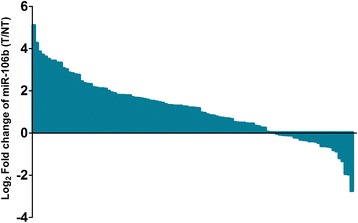
**Upregulation of miR-106b was observed in 79/104 (76.0%) HCCs.**

We performed quantitative real-time RT-PCR to examine the miR-106b expression level in 104 pairs of HCC and adjacent non-tumor tissues. As shown in Figure 1, the expression level of miR-106b in HCC tissues was significantly higher than that in adjacent non-tumor tissues after normalization (median fold change of T/NT = 2.27, *P* < 0.001). MiR-106b was upregulated in 79 of 104 patients (76.0%) totally (Figure 2). The median fold change of miR-106b was used as a cutoff value to divide all 104 patients into two groups: the low expression group (n = 52) and the high expression group (n = 52).

### Correlation of miR-106b expression with clinicopathological characteristics of HCC patients

**Figure 3:**
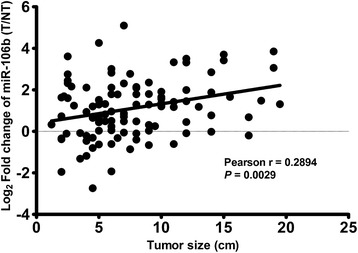
**MiR-106b expression correlated with tumor size.** A Pearson correlation analysis showed that the miR-106b level and tumor size were positively correlated (r = 0.2894, *P* = 0.0029).

We next analyzed the correlation between miR-106b expression and the clinicopathological characteristics of HCC, including patients’ age, gender, HBsAg, Child-Pugh classification, serum AFP level, tumor size, tumor number, vascular invasion, histological grade (Edmondson-Steiner) and TNM stage. As summarized in Table 1, miR-106b expression was significantly higher in HCC patients with large tumor than those with small tumor (*P* = 0.019). Additionally, a Pearson correlation analysis also showed that the miR-106b level and tumor size were positively correlated (r = 0.2894, *P* = 0.0029; Figure 3). Moreover, miR-106b was expressed at significantly higher levels in patients with vascular invasion than in patients without vascular invasion (*P* = 0.016). However, no significant correlation was observed between miR-106b expression and other clinicopathological characteristics.

**Table 1:** **Correlation between relative miR-106b expression and clinicopathological characteristics in HCCs (n = 104)**

**Characteristics**	**No. of cases**	**miR-106b**		***P*** **value** ^**a**^
		**Low**	**High**	
Age (years)				
<50^b^	49	22 (44.9)	27 (55.1)	0.326
≥50	55	30 (54.5)	25 (45.5)	
Gender				
Female	10	3 (30.0)	7 (70.0)	0.183
Male	94	49 (52.1)	45 (47.9)	
HBsAg				
Negative	18	12 (66.7)	6 (33.3)	0.120
Positive	86	40 (46.5)	46 (53.5)	
Child-Pugh				
A	95	48 (50.5)	47 (49.5)	>0.999
B	9	4 (44.4)	5 (55.6)	
AFP (μg/L)				
<200^c^	52	30 (57.7)	22 (42.3)	0.117
≥200	52	22 (42.3)	30 (57.7)	
Tumor size (cm)				
<7^d^	50	31 (62.0)	19 (38.0)	**0.019**
≥7	54	21 (38.9)	33 (61.1)	
Tumor number				
Solitary	70	33 (47.1)	37 (52.9)	0.403
Multiple	34	19 (55.9)	15 (44.1)	
Vascular invasion				
Absent	82	46 (56.1)	36 (43.9)	**0.016**
Present	22	6 (27.3)	16 (72.7)	
Edmondson-Steiner				
I-II	60	34 (56.7)	26 (43.3)	0.112
III-IV	44	18 (40.9)	26 (59.1)	
TNM stage				
I	51	27 (52.9)	24 (47.1)	0.556
II-III	53	25 (47.2)	28 (52.8)	

^a^Chi-square or Fisher’s exact test.

^b,c,d^Values are median.

Values in parentheses indicate percentage.

Statisticaly significant values are given in bold.

### Prognostic analysis of miR-106b expression and clinicopathological factors

**Figure 4:**
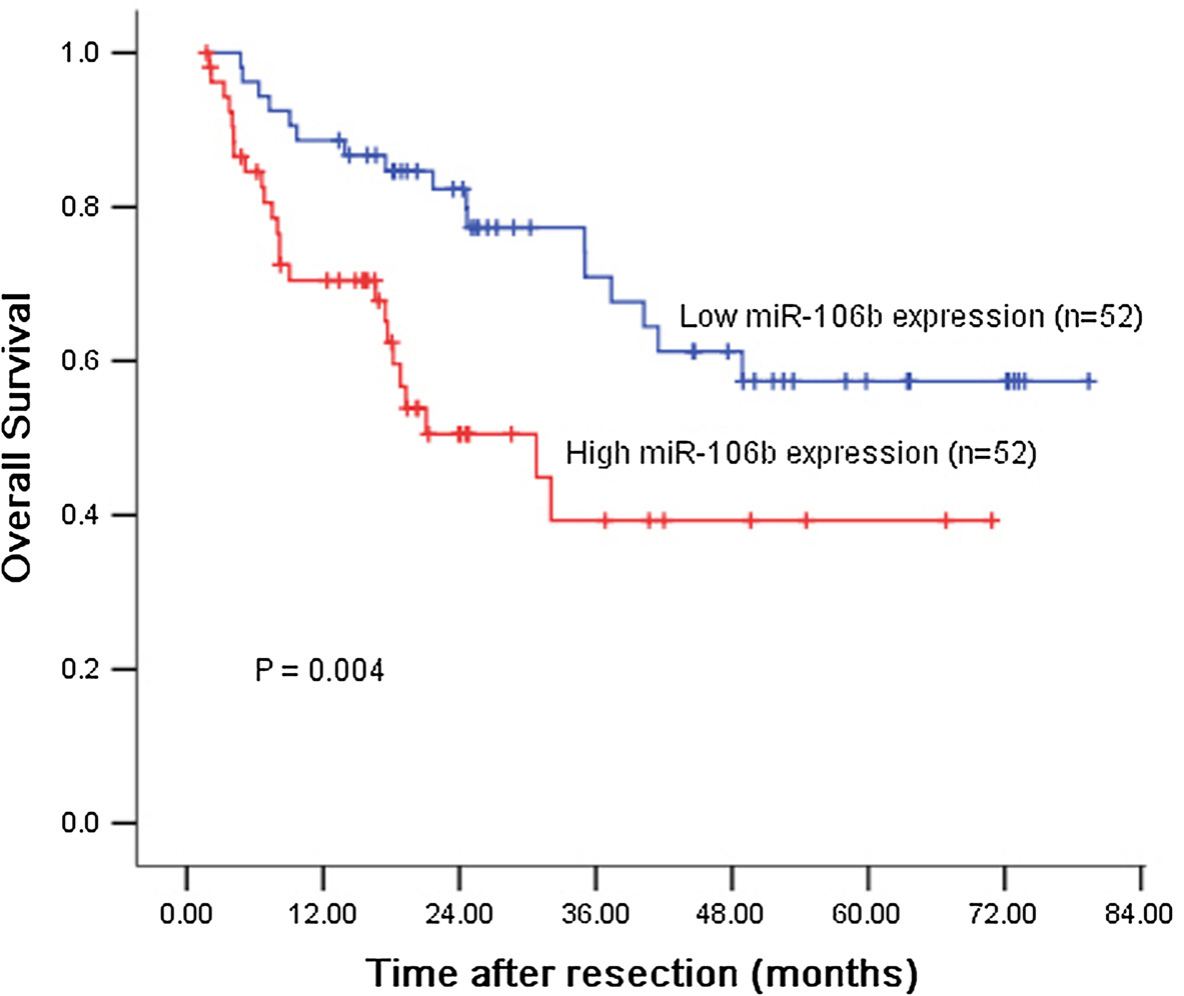
**Survival analysis of 104 HCC patients by Kaplan-Meier method.** Overall survival rate in patients with high miR-106b expression was significantly lower than that in patients with low miR-106b expression (log-rank *P* = 0.004).

The association between miR-106b expression and prognosis of HCC patients was investigated by Kaplan-Meier analysis and log-rank test. As shown in Figure 4, HCC patients with high miR-106b expression had shorter overall survival than those with low miR-106b expression. The 1, 3, and 5-year overall survival rate in the high expression group was 84.0%, 51.6%, and 36.5%, respectively, compared with 84.4%, 60.2%, and 56.2%, respectively, in the low expression group (log-rank test, *P* = 0.004).

Univariate analysis demonstrated that Serum AFP level (*P* = 0.041), tumor size (*P* < 0.001), vascular invasion (*P* = 0.001), histological grade (*P* = 0.048) and TNM stage (*P* = 0.004), and miR-106b expression (*P* = 0.004) were significantly associated with overall survival of HCC patients (Table 2). No significant associations were found for age at diagnosis, gender, HBsAg status, Child-Pugh classification, and tumor number. Multivariate analysis using the Cox proportional hazards model for variables that were significant in the univariate analysis showed that tumor size (*P* = 0.022), vascular invasion (*P* = 0.028) and miR-106b expression (*P* = 0.027) were independent prognostic factors for patients with HCC (Table 2).

**Table 2:** **Univariate and multivariate Cox regression analyses for overall survival**

**Variable**	**Univariate analysis**		**Multivariate analysis**	
	**HR (95% CI)**	***P***	**HR (95% CI)**	***P***
miR-106b (high versus low)	2.445 (1.299-4.605)	**0.004**	2.002 (1.130-6.977)	**0.027**
Age (≥50 versus <50 years)	1.295 (0.691-2.426)	0.419	-	-
Gender (M versus F)	1.195 (0.368-3.874)	0.767	-	-
HBsAg (positive versus negative)	1.980 (0.774-5.066)	0.154	-	-
Child-Pugh (B versus A)	2.027 (0.790-5.199)	0.141	-	-
AFP (≥200 versus <200 μg/L)	1.926 (1.027-3.612)	**0.041**	1.263 (0.650-2.453)	0.491
Tumor size (≥7 cm versus <7 cm)	3.435 (1.746-6.757)	**<0.001**	2.353 (1.133-4.884)	**0.022**
Tumor number (multiple versus solitary)	1.745 (0.941-3.235)	0.077	-	-
Vascular invasion (present versus absent)	2.927 (2.083-7.403)	**0.001**	2.340 (1.001-5.466)	**0.028**
Edmondson-Steiner (III-IV versus I-II)	2.219 (1.006-4.895)	**0.048**	1.303 (0.534-3.180)	0.369
TNM stage (II-III versus I)	2.331 (1.391-5.359)	**0.004**	1.234(0.398-3.822)	0.716

Statisticaly significant values are given in bold.

## Discussion and conclusions

MiRNAs are considered to be ideal biomarkers for the diagnosis and prognosis of cancer because they are easy to detect and strongly associated with clinical prognoses [25-27]. So far, increasing evidence shows that more and more deregulated miRNAs are involved in HCC carcinogenesis and patient prognosis [7,8,20-23,28]. However, due to the complicated and diverse roles of miRNAs in HCC, clarifying the clinical significance and function of an individual miRNA may enable deeper insights into the complexity of HCC progression and disease management.

MiR-106b is a member of the oncogenic miR-106b-25 cluster [16]. The biological function of miR-106b in HCC has been investigated in previous studies. It has been demonstrated that ectopic expression of miR-106b can enhance the proliferation and anchorage-independent growth of HCC cells, whereas inhibition of miR-106b had the opposite effect [20,22]. It has also been shown that miR-106b contributed to metastasis by activating the EMT process and promoting cell migration in vitro and metastasis in vivo in HCC, indicating that upregulation of miR-106b may correlate with HCC progression [21]. However, the clinical significance and prognostic value of miR-106b in HCC remain unclear.

In the present study, we analyzed the clinical relevance of miR-106b expression in HCC patients. Our qRT-PCR data confirmed that miR-106b expression was upregulated in tumor tissues compared with the adjacent non-tumor tissues, which validated our previous microarray high-throughput profiling results [23]. These results are also consistent with previous findings in other studies [20,21]. In addition, we correlated clinicopathological characteristics of the patients with miR-106b expression and observed that overexpression of miR-106b correlated with large tumor size and vascular invasion, which strongly indicated that this miRNA plays oncogenic roles in HCC, including promoting cell growth, cell invasion and tumor metastasis.These observations could be attributed to the miR-106b target genes, which include p21/CDKN1A, adenomatous polyposis coli (APC), transforming growth factor-β type II receptor (TGF-βRII), and RhoA and RhoC [16,21,22,29]. Hence, by regulating its target genes, miR-106b could promote HCC cell cycle progression, cell proliferation, and cell migration and invasion.

More importantly, we demonstrated that miR-106b expression was significantly associated with overall survival of patients with HCC. Kaplan-Meier analysis showed that patients with high miR-106b expression level had a significantly shorter overall survival than those with low miR-106b expression level. Furthermore, multivariate Cox analysis proved that miR-106b was a prognostic factor independent of adjusted well-known prognostic variables for HCC including serum AFP level, tumor size, vascular invasion, histological grade, and TNM stage. Thus, miR-106b could be used as a potential prognostic biomarker in addition to other known prognostic indicator, in order to identify a subgroup of patients who have higher risk of death, thus, should receive monitoring more frequently and effective adjuvant treatment. Many studies have shown that miRNA profiles are maintained and are not significantly affected in formalin-fixed paraffin-embedded tissues [30,31]. Therefore, although our study is based on frozen tissue samples, it offers a significant opportunity for using archived samples to detect changes in miR-106b expression levels by using in situ hybridization methodology with miRNA localization in routine clinical setting [32]. In summary, our data indicated that miR-106b expression was significantly upregulated and associated with poor prognosis in HCC. MiR-106b was identified as an independent biomarker for predicting the clinical prognosis of HCC patients. Further studies are needed to validate the prognostic value of miR-106b expression in other cohorts.

## Abbreviations

miRNA: MicroRNA
HCC: Hepatocellular carcinoma
qRT-PCR: Quantitative real-time PCR
MCM7: Minichromosome maintenance complex component 7
AFP: Alpha-fetoprotein
HR: Hazard ratio
APC: Adenomatous polyposis coli
TGF-βRII: Transforming growth factor-β type II receptor
